# HLA-associated viral polymorphism in chronically HIV-1-infected Japanese cohort: analysis of four-digit HLA allele level

**DOI:** 10.1186/1742-4690-9-S2-P269

**Published:** 2012-09-13

**Authors:** T Chikata, JM Carlson, Y Tamura, ZL Brumme, T Naruto, M Hashimoto, MA Borghan, M John, S Mallal, H Gatanaga, S Oka, M Takiguchi

**Affiliations:** 1 Center for AIDS Research, Kumamoto University, Kumamoto, Japan; 2 eScience Group, Microsoft Research, Redmond, WA, USA; 3 Faculty of Health Sciences, Simon Fraser University, Burnaby, British Columbia, Canada; 4 College of Arts and Sciences, University of Nizwa, Oman; 5 Royal Perth Hospital, Murdoch University, Perth, Australia; 6 AIDS Clinical Center, National Center for Global Health and Medicine, Tokyo, Japan

## Background

It is assumed that the difference of HLA class I distribution among ethnic populations influences HIV evolution because HLA-restricted immune pressure selects escape mutations. Approximately 50% of HLA class I alleles are shared between Japanese and Caucasians. The analysis of HLA-associated polymorphism (HLA-AP) in both Japanese and Caucasian infected with clade B virus is expected to clarify the difference of HIV-1 evolution between both populations.

## Methods

We sequenced Gag, Pol, and Nef genes in 430 treatment-naïve Japanese chronically infected with HIV-1 clade B and then identified HLA-associated amino acids at each codon using a phylogenetically corrected logistic regression model and false discovery rates to correct for multiple tests.

## Results

We completely determined 400, 366, and 309 sequences of Gag, Pol, and Nef, respectively, and then analyzed polymorphisms associated with 78 four-digit HLA alleles (21 HLA-A, 38 HLA-B, and 19 HLA-C alleles). At the threshold of q<0.2, we found 195 HLA-APs (67 in Gag, 61 in Pol, and 67 in Nef). These polymorphisms were observed at 39 of 501 (7.8%) Gag, 42 of 1004 (4.2%) Pol, and 33 of 207 (16.0%) Nef codons. Ninety-six HLA-APs associated with more than one HLA subtype allele were detected in 4-digital HLA allele analysis. Approximately 40% of HLA-APs were associated with HLA alleles predominantly found in Asia. Out of HLA-APs associated with common HLA alleles, approximately 50% were found in Caucasian population (IHAC cohort).

## Conclusion

This study demonstrated that only 30% of HLA-APs were shared between Caucasians and Japanese, indicating that the difference in HLA allele distributions resulted in distinct HIV-1 evolution.

